# An unusual cause of gastric outlet obstruction during percutaneous endogastric feeding: a case report

**DOI:** 10.1186/1752-1947-2-199

**Published:** 2008-06-11

**Authors:** Abdulzahra Hussain, Hind Mahmood, Tarun Singhal, Shamsi El-Hasani

**Affiliations:** 1 General Surgery Department, Princess Royal University Hospital, Kent, UK

## Abstract

**Introduction:**

The differential diagnoses of acute abdomen in children include common and rare pathologies. Within this list, different types of bezoars causing gastrointestinal obstruction have been reported in the literature and different methods of management have been described. The aim of this article is to highlight a rare presentation of lactobezoars following prolonged percutaneous endoscopic gastrostomy feeding and its successful surgical management.

**Case presentation:**

A 16-year-old boy was admitted to a paediatric ward with abdominal distension and high output from his permanent gastrostomy feeding tube, with drainage of bilious fluids. The clinical, radiological and endoscopical examinations were suggestive of partial duodenal obstruction with multiple bezoars in the stomach and duodenum. Gastrojejunostomy was performed after the removal of 14 bezoars. The child had an uneventful postoperative course and was discharged on the sixth postoperative day in a stable condition.

**Conclusion:**

Lactobezoars should be included in the differential diagnosis of acute abdominal pain in patients with percutaneous endogastric feeding. Endoscopy is important in making the diagnosis of this surgical condition of the upper gastrointestinal tract in a child.

## Introduction

Clinical assessment of acute abdomen in children poses a challenge to both the paediatrician and the surgeon. Foreign bodies are one of the main causes of acute abdomen in children. In general, most upper gastrointestinal (GI) tract foreign bodies are related to food impaction, with meat being the most frequent culprit [1]. Bezoars occur most commonly in patients with impaired GI motility or a history of gastric surgery [2]. While gastric bezoars are rare, and usually observed in female children with mental or emotional disorders [3], other parts of the GI tract may be affected. Recent significant advances in imaging technology have changed the approach and algorithm of management of many bezoar emergencies [4], but successful management is usually achieved by endoscopy and surgery. Here we present a rare case of lactobezoars and the role of endoscopy, laparoscopy and surgery in the management.

## Case presentation

A 16-year-old boy was admitted to a paediatric ward because of abdominal distension and a high output from his percutaneous endogastric (PEG) tube, with drainage of bilious fluids. He had been admitted twice over the last 6 months because of abdominal distension and constipation, and had been treated conservatively with intravenous fluids and enemas and had responded well.

His past medical history was suggestive of cerebral palsy and convulsions. He had a significant surgical history of a ventriculo-peritoneal shunt, Nissen anti-reflux surgery, and insertion of a PEG tube at the age of 4 years.

**Figure 1:**
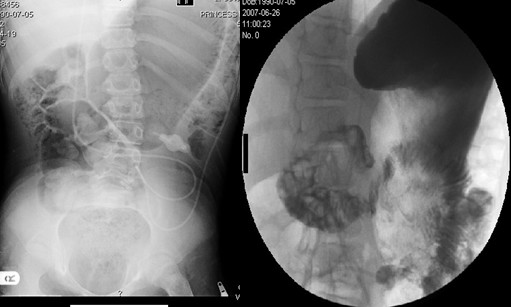
Plain abdomen X-ray and gastrografin studies.

**Figure 2:**
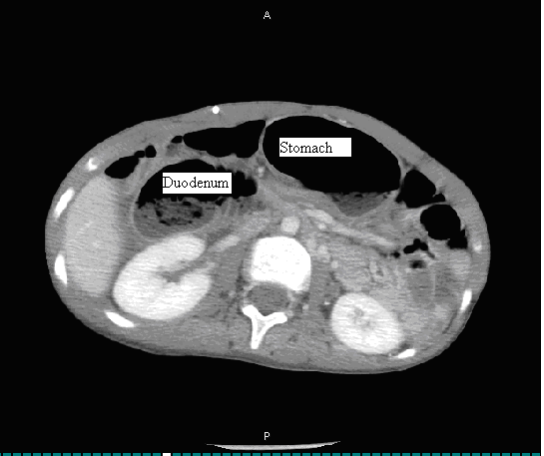
Abdominal computed tomography scan shows dilated stomach, duodenum and duodenal stenosis.

**Figure 3:**
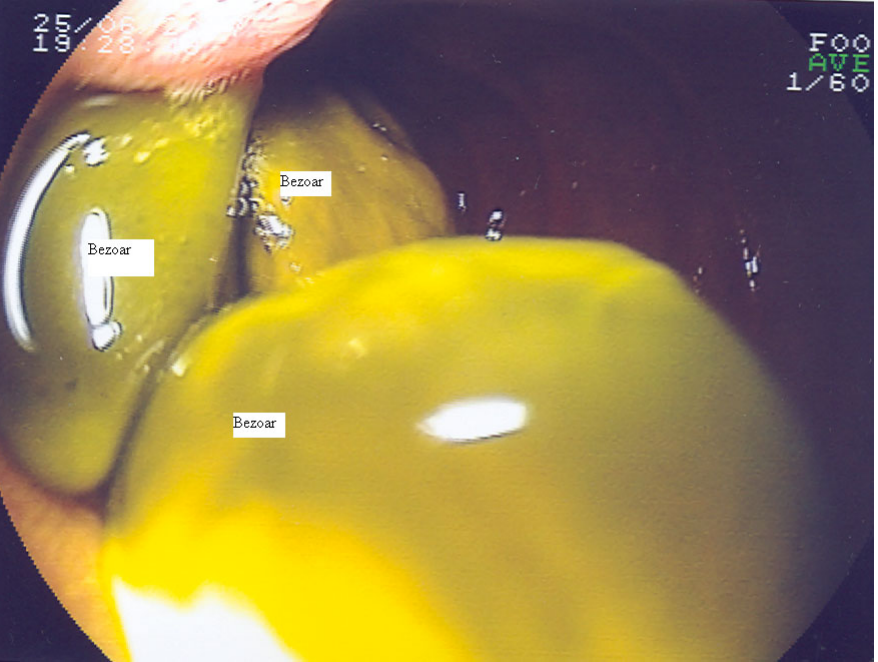
Endoscopic findings of the third part of the duodenum showing multiple bezoars.

Clinical and radiological examinations indicated incomplete duodenal obstruction (see figures 1, 2, 3). Oesophago-gastro-duodenoscopy confirmed gastric and duodenal dilatation secondary to obstruction by multiple bezoars in the stomach and duodenum. Laparoscopy was considered risky because of extensive adhesions from previous laparotomies. Release of adhesions and an antecolic posterior gastrojejunostomy were performed after removal of 14 lactobezoars. The patient's postoperative course was uneventful.

## Discussion

A bezoar is a concretion of foreign material in the GI tract. Depending on the material contained within, they may be trichobezoars, phytobezoars, lactobezoars or others. Phytobezoars are more common, while trichobezoars are rare. Common predisposing factors are previous gastric surgery, psychiatric illness, coeliac disease and metabolic disorders such as uraemia [5].

Recurrent abdominal pain or acute small bowel obstruction is the usual presentation of a GI bezoar. A history of foreign body ingestion, especially in children and mentally impaired patients, is important [6]. Rarely, bezoars can cause serious problems due to complications such as perforation [7]. Endoscopy and radiological studies, including ultrasound, computed tomography scan and gastrografin swallow, may help make the diagnosis.

A range of methods have been used in the management of bezoars. These include endoscopy, surgery, combined laparoscopy and surgery, and the use of emulsifying chemical materials. In uncomplicated cases, endoscopic or surgical removal can be appropriate [8]. For our patient we planned laparoscopic exploration and possible adhesiolysis and laparoscopic gastrojejunostomy. However, it was difficult to proceed with laparoscopic management because of the extensive adhesions caused by previous surgery. Laparotomy confirmed the endoscopic and radiological findings of massive distension of the stomach and duodenum in addition to the adhesions. There was no definite extrinsic cause for duodenal stenosis apart from the adhesions, which were released. Antecolic posterior gastrojejunostomy was performed after removal of 14 lactobezoars (1 × 1.5 cm each). The patient responded very well and his postoperative course was unremarkable.

## Conclusion

Lactobezoars should be included in the differential diagnosis of acute abdomen in children with PEG feeding. Early surgical assessment is important in the management of this condition. Endoscopy in children can be important in the diagnosis of surgical conditions of the upper GI tract.

## Competing interests

The authors declare that they have no competing interests.

## Consent

Written informed consent was obtained from the patient's next-of-kin for publication of this case report and accompanying images. A copy of the written consent is available for review by the Editor-in-Chief of this journal.

## Authors' contributions

AH wrote the article, participated in the sequence alignment and drafted the manuscript, HM participated in the sequence alignment, formatted the pictures and performed language corrections, TS collected the data and investigation studies, participated in the article design and critically evaluated the article, SEH conceived the study, and participated in its design and coordination and helped to draft the manuscript. All authors read and approved the final manuscript.
